# Dystonia 3×3: A Training Guideline for Beginners in Botulinum Neurotoxin Treatment of Cervical Dystonia

**DOI:** 10.3390/toxins18090378

**Published:** 2026-09-02

**Authors:** Sebastian Paus, Andreas Funke, Jürgen Hamacher, Bernhard Haslinger, Jan Heckelmann, Chi Wang Ip, Wolfgang H. Jost, Anatol Kivi, Stephan Klebe, John-Ih Lee, Ebba Lohmann, Frederic Mack, Axel Schramm, Andrea Stenner, Pawel Tacik, Uwe Walter, David T. Weise

**Affiliations:** 1 Department of Neurology, GFO Kliniken Troisdorf, 53844 Troisdorf, Germany; 2 Center for Neurology, University Hospital Bonn, 53127 Bonn, Germany; 3 Neurologie am Funkerberg, 15711 Königs Wusterhausen, Germany; 4 Praxis NeuroMotion, 45219 Essen, Germany; 5 Department of Neurology, Technical University Munich, 81675 Munich, Germany; 6 Department of Neurology, RWTH University Hospital Aachen, 52074 Aachen, Germany; 7 Department of Neurology, University Hospital Würzburg, 97080 Würzburg, Germany; 8 Parkinson-Klinik Ortenau, 77709 Wolfach, Germany; 9 Department of Neurology, Saarland University, 66421 Homburg, Germany; 10 Department of Neurological Rehabilitation and Physical Therapy, Vivantes Klinikum Spandau, 13585 Berlin, Germany; 11 Department of Neurology, Knappschaft Kliniken Recklinghausen, 45657 Recklinghausen, Germany; 12 Department of Neurology and Clinical Neurophysiology, GFO Kliniken Rhein-Berg, 51465 Bergisch Gladbach, Germany; 13 Department of Neurology, Medical Faculty, University Hospital Düsseldorf, Heinrich Heine University Düsseldorf, 40225 Düsseldorf, Germany; 14 Department of Neurology and Hertie Institute for Clinical Brain Research, University of Tübingen, 72076 Tübingen, Germany; 15 Deutsches Zentrum für Neurodegenerative Erkrankungen (DZNE), University of Tübingen, 72076 Tübingen, Germany; 16 NeuroPraxis Fuerth, 90762 Fuerth, Germany; 17 Movement Disorders Competence Center, Medizinisches Versorgungszentrum Zwickau, 08060 Zwickau, Germany; 18 Center for Neurology, Department of Parkinson’s Disease, Sleep and Movement Disorders, University Hospital Bonn, 53127 Bonn, Germany; 19 Department of Neurology, Rostock University Medical Center, 18147 Rostock, Germany; 20 Deutsches Zentrum für Neurodegenerative Erkrankungen (DZNE) Rostock/Greifswald, 18147 Rostock, Germany; 21 Department of Neurology, Asklepios Fachklinikum Stadtroda, 07646 Stadtroda, Germany; 22 Department of Neurology, University of Leipzig, 04103 Leipzig, Germany

**Keywords:** cervical dystonia, botulinum neurotoxin type A, training concept, ultrasound, Col-Cap-Concept, expert consensus

## Abstract

Cervical dystonia (CD) is a hyperkinetic movement disorder characterized by involuntary muscle contractions leading to abnormal postures of the head and neck. The injection of botulinum neurotoxin type A (BoNT-A) into the muscles involved is the treatment of choice for CD. BoNT-A therapy of CD requires appropriate training, which has not been standardized in Germany. To address the key challenges in BoNT-A education, an expert panel developed a simplified step-by-step treatment guideline for beginners, adapted from the established Col-Cap-Concept (“Dystonia 3×3”). Based on expert consensus, recommendations were developed for the core clinical elements of BoNT-A treatment in CD—recognition of the movement pattern, identification of target muscles, development of an injection plan, ultrasound-guided injection, and dosing, taking into account the target audience of the guideline. As such, Dystonia 3×3 provides a user-friendly beginner’s guidebook, enhancing accessibility and standardization of CD treatment training. Further research is required to evaluate its clinical efficacy and suitability for training.

## 1. Introduction

Cervical dystonia (CD) is a chronic neurological hyperkinetic movement disorder. It is characterized by persistent or intermittent involuntary contractions of cervical muscles, causing atypical postures of the head, neck, and/or shoulders, often accompanied by tremor and pain [1,2]. The injection of botulinum neurotoxin type A (BoNT-A) into the muscles involved in the movement disorder is the treatment of choice in CD [3]. In Europe and the US, AboBoNT-A (Dysport^®^), OnaBoNT-A (BOTOX^®^), and IncoBoNT-A (XEOMIN^®^) have been approved, while DaxiBoNT-A (DAXXIFY^®^) is approved in the US only [4,5]. Numerous studies have demonstrated BoNT-A to be effective and generally well tolerated [6]. BoNT-A treatment of CD follows a multi-stage approach: movement analysis and muscle identification are pivotal factors that influence treatment efficacy [7]. This is followed by the selection of toxin, dilution, and dosing [7]. Locating the muscle can be facilitated by ultrasound and/or electromyography, which enables more precise injection [7,8].

Examining and defining movement disorders of the head and neck is often challenging. To help identify the muscles involved in distinct patterns of dystonic movements, a detailed classification of CD movements known as the Col-Cap-Concept was presented in 2011 [9] and later refined [8,9]. This classification is based on the differentiation of movements of the head (caput) and the cervical spine (collis). Then, the direction of movements is considered: lateral tilt (latero-), rotation (torti-), anteflexion (antero-), and retroflexion (retro-) [9]. This classification results in eight basic patterns of CD movements: laterocaput, laterocollis, torticaput, torticollis, anterocaput, anterocollis, retrocaput, and retrocollis. However, in more than 60% of CD patients, combinations of these basic patterns are prevalent, including horizontal lateral or sagittal movements (shift) [9]. Furthermore, the complexity of treatment is increased by tremor and pain [7,8]. In total, the Col-Cap-Concept incorporates 14 muscles, treated either unilaterally or bilaterally [8,9].

In recent years, challenges in the training of CD treatment have emerged, emphasizing the importance of a structured, step-by-step approach for teaching BoNT-A application in movement disorders. Furthermore, there is no standardized or validated training method for beginners in CD treatment, at least in Germany. To develop a simplified yet holistic training method for the clinical and technical elements of CD treatment, a board of movement disorder specialists experienced in CD followed a structured procedure based on the Col-Cap-Concept and its refinements. First, movement patterns of CD eligible for treatment by beginners, and the pivotal muscles involved in each, were identified. Second, ultrasound identification and doses for each muscle were determined, resulting in a structured and user-friendly beginners’ guidebook for CD treatment (“Dystonia 3×3”).

We would like to emphasize that the guideline presented here is intended to teach the core clinical competencies involved in BoNT treatment of CD. It was developed as a training tool for physicians (as prerequisite qualification) who have no or only little previous experience in treating patients with CD (“beginners”). Numerous additional aspects of CD patient care—including diagnosis, differential diagnosis, patient education, BoNT preparation, potential treatment-related side effects, oral and surgical treatment options, non-motor symptoms and long-term management—are not covered by Dystonia 3×3. These skills need to be learned and taught in practical courses, ideally involving affected patients. Dystonia 3×3 cannot and should not replace these trainings but, rather, is intended to provide a low-threshold introduction to various elements of CD treatment that are related to the motor syndrome.

## 2. Results

Of the various elements of BoNT-treatment of CD, the expert panel defined five consecutive steps as pivotal for the training of beginners: (1) the definition of movement patterns eligible for beginners, (2) target muscle identification, (3) the relevance of ultrasound visualization and placement of the ultrasound head, (4) the development of an injection plan for CD patients, and (5) the determination of the appropriate BoNT-A dose, according to preparation. Consensus was reached for all results in these five areas.

**Figure 1:**
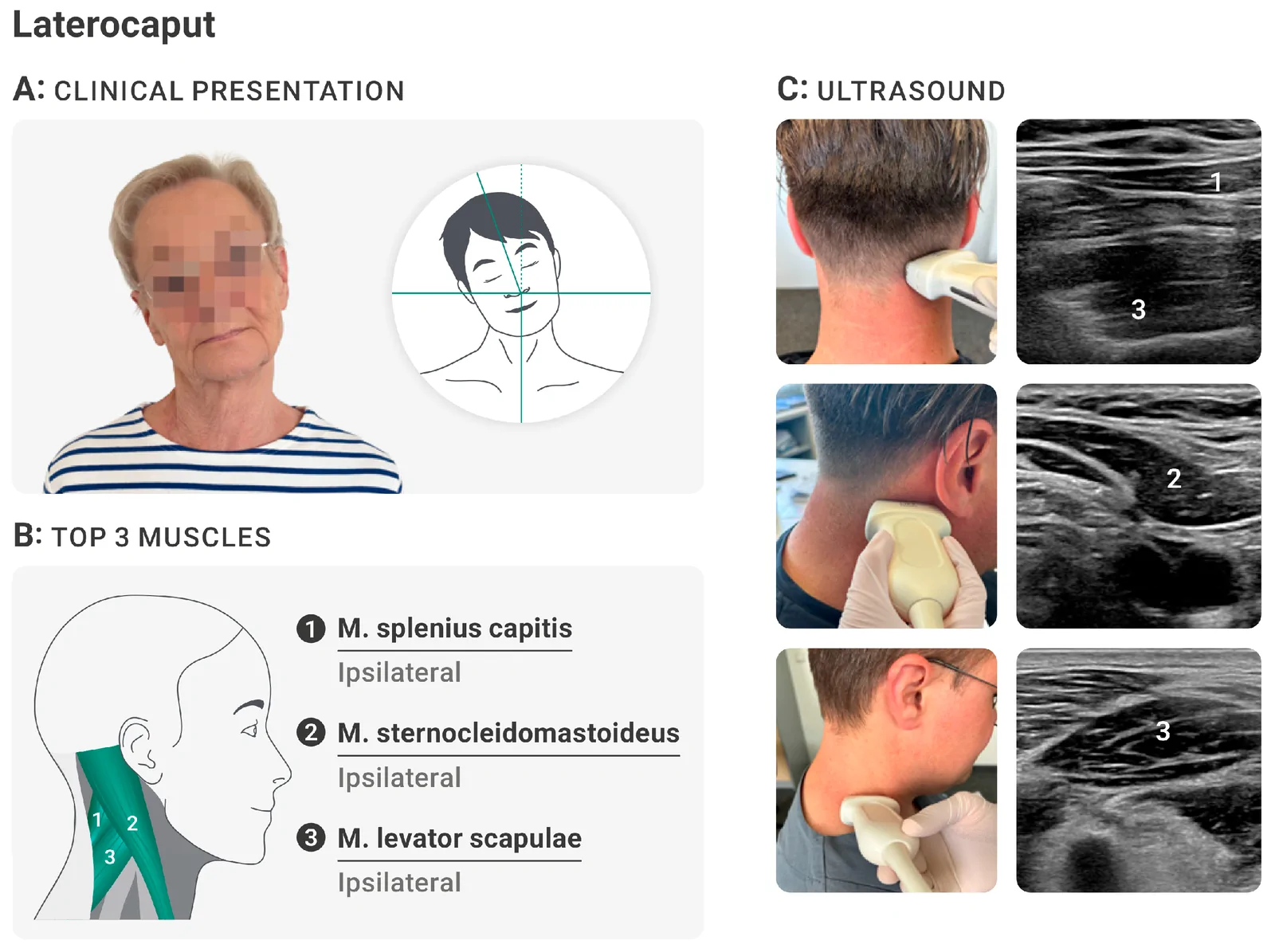
Laterocaput: Clinical presentation, TOP 3 muscles, and ultrasound visualization. (**A**) Depicted is the lateral tilt of the head (caput) relative to the neck (collum) in laterocaput. (**B**) The three most important muscles to be injected are m. splenius capitis (1), m. sternocleidomastoideus (2), and m. levator scapulae (3). All are injected ipsilateral to the head tilt. (**C**) Positioning of the ultrasound head (left) and ultrasound images (right) of the respective muscles are shown (1: m. splenius capitis; 2: m. sternocleidomastoideus; 3: m. levator scapulae).

**Figure 2:**
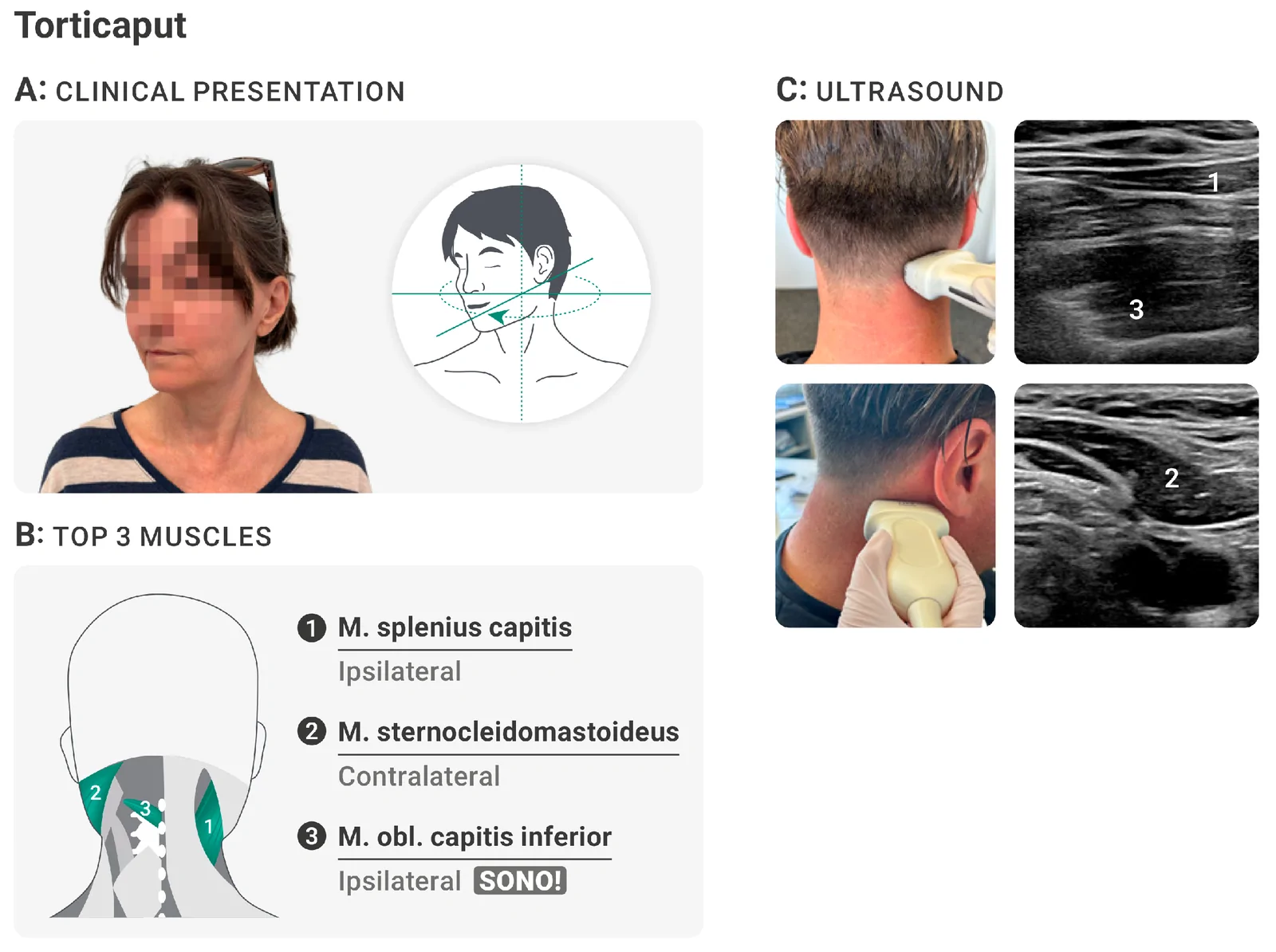
Torticaput: Clinical presentation, TOP 3 muscles, and ultrasound visualization. (**A**) Depicted is the rotation of the head (caput) relative to the neck (collum) in torticaput. (**B**) The three main muscles to be injected are m. splenius capitis (1), m. sternocleidomastoideus (2), and m. obl. capitis inferior (3). M. sternocleidomastoideus is injected contralateral to the rotation, while m. splenius capitis and m. obl. capitis inferior are injected ipsilaterally to the rotation. Especially in m. obl. capitis inferior, injection under ultrasound guidance is mandatory (SONO!). (**C**) Positioning of the ultrasound head (left) and ultrasound images (right) of the respective muscles are shown (1: m. splenius capitis; 2: m. sternocleidomastoideus; 3: m. obl. capitis inferior).

**Figure 3:**
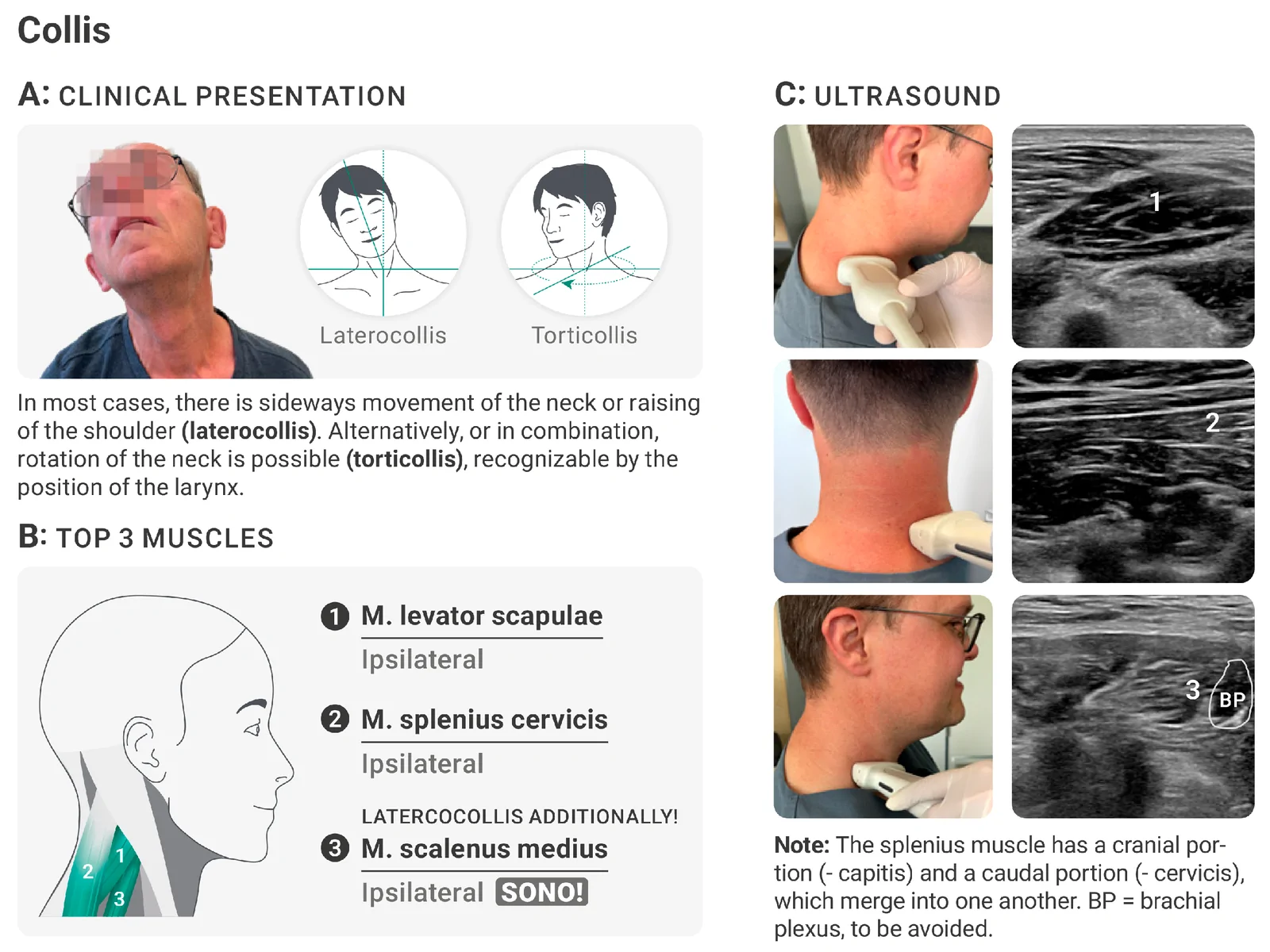
Collis: Clinical presentation, TOP 3 muscles, and ultrasound visualization. (**A**) The photograph depicts a combined lateral tilt and rotation of the neck relative to the torso, representing a mixed laterocollis–torticollis movement. (**B**) The most important muscles to be injected are m. levator scapulae (1) and m. splenius cervicis (2), in addition to m. scalenus medius (3) in laterocollis. All are injected ipsilaterally to the head tilt or rotation. Especially in m. scalenus medius, injection under ultrasound guidance is mandatory (SONO!). (**C**) Positioning of the ultrasound head (left) and ultrasound images (right) of the respective muscles are shown (1: m. levator scapulae; 2: m. splenius cervicis; 3: m. scalenus medius).

**Figure 4:**
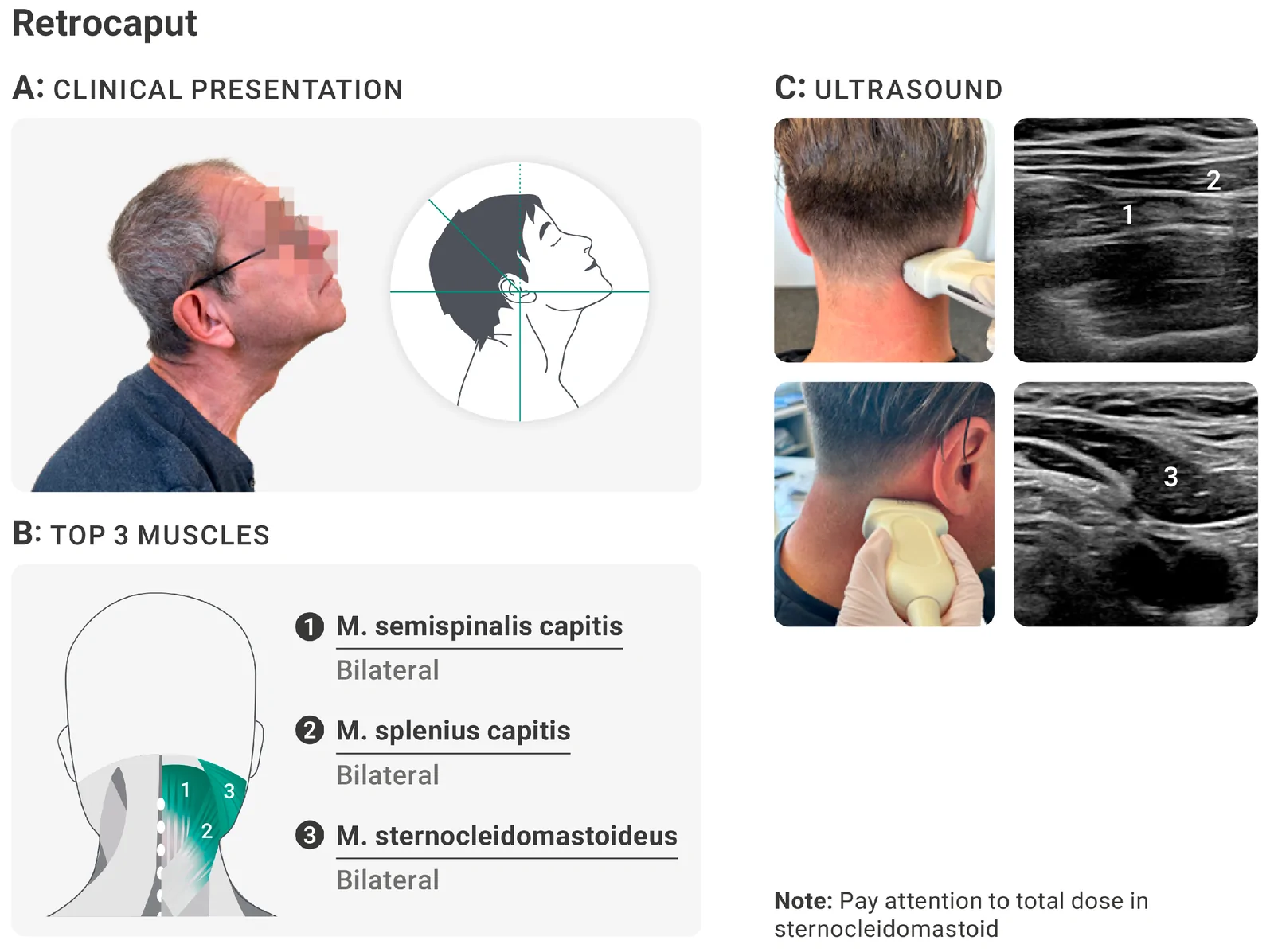
Retrocaput: Clinical presentation, TOP 3 muscles, and ultrasound visualization. (**A**) Depicted is the posterior hyperextension of the head (caput), while the neck (collum) remains largely neutral. (**B**) The three most important muscles to be injected are m. semispinalis capitis (1), m. splenius capitis (2), and m. sternocleidomastoideus (3). All are injected bilaterally. (**C**) Positioning of the ultrasound head (left) and ultrasound images (right) of the respective muscles are shown (1: m. semispinalis capitis; 2: m. splenius capitis; 3: m. sternocleidomastoideus).

**Figure 5:**
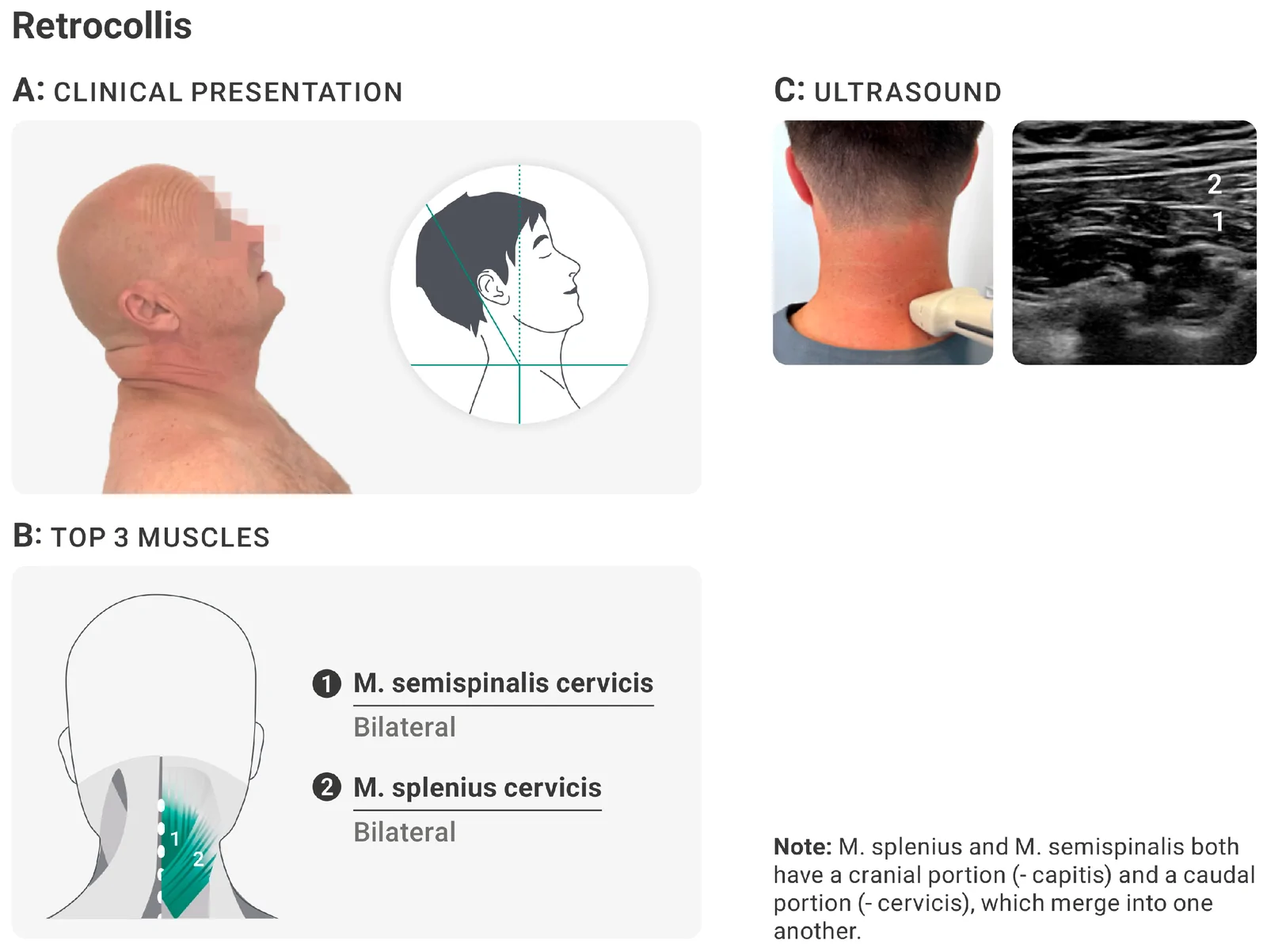
Retrocollis: Clinical presentation, TOP 3 muscles, and ultrasound visualization. (**A**) Depicted is the hyperextension of the neck (caput) backwards relative to the torso. (**B**) The two most important muscles to be injected are m. semispinalis cervicis (1) and m. splenius cervicis (2). Both are injected bilaterally. (**C**) Positioning of the ultrasound head (left) and ultrasound images (right) of the respective muscles are shown (1: m. semispinalis cervicis; 2: m. splenius cervicis).

**Figure 6:**
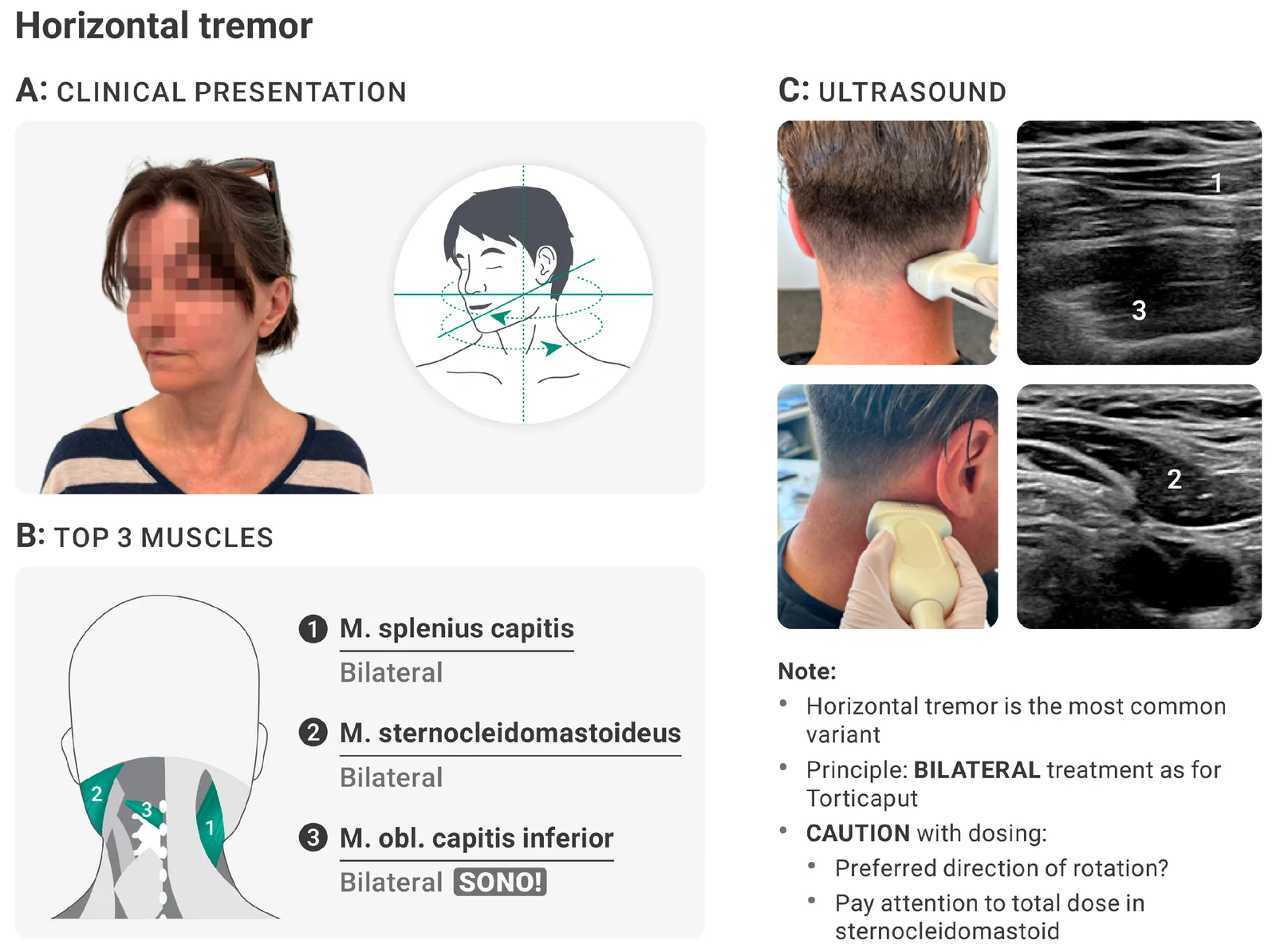
Horizontal tremor: Clinical presentation, TOP 3 muscles, and ultrasound visualization. (**A**) Depicted is the horizontal tremor of the head (caput) relative to the torso. (**B**) The three most important muscles to be injected are m. splenius capitis (1), m. sternocleidomastoideus (2), and m. obl. capitis inferior (3). All are injected bilaterally. (**C**) Positioning of the ultrasound head (left) and ultrasound images (right) of the respective muscles are shown (1: m. splenius capitis; 2: m. sternocleidomastoideus; 3: m. obl. capitis inferior). Especially in m. obl. capitis inferior, injection under ultrasound guidance is mandatory (SONO!).

For the maximal user-friendliness and bedside practicability of the guideline, clinical presentation, including patient examples (A), associated muscles (B), and placement of the ultrasound head and ultrasound visualization (C), were condensed into single-page graphical representations for each CD movement pattern (Figure 1, Figure 2, Figure 3, Figure 4, Figure 5 and Figure 6). Some comments were also added as notes.

### 2.1. CD Movement Patterns

A total of six movement patterns were identified as suitable for beginners in the BoNT-treatment of CD: laterocaput, torticaput, collis, retrocaput, retrocollis, and horizontal tremor (Table 1 and Figure 1, Figure 2, Figure 3, Figure 4, Figure 5 and Figure 6, respectively). The anterior CD variants, anterocollis and anterocaput, were not included in Dystonia 3×3 [10,11]. In these CD patterns, associated muscles include longus capitis and longus colli, located deeper in the neck and close to major blood vessels. Due to this anatomical complexity and associated risks, injection placement is more difficult [12]. Despite recent advancements in injection techniques [13], both muscles and associated dystonic patterns were determined as ineligible for beginners and therefore omitted from this guideline.

For the training of beginners, the panel established the “collis” pattern as an aggregation of laterocollis and torticollis (Figure 3). In clinical practice, the differentiation of the two movements is usually impractical, and both often merge. Additionally, the associated muscles are largely identical, with the scalenus medius being specifically associated with laterocollis.

A guideline for approaching the treatment of frequently encountered mixed-pattern CD patients is suggested in the injection plan (see below).

### 2.2. Identification of Associated Muscles

In the literature, there is insufficient information on the number of muscles involved in the various dystonic patterns of CD. However, there was consensus that the treatment of a limited number of muscles for the core movements of Dystonia 3×3 is sufficient for the initial approach in novice patients. As such, the determination of associated muscles was limited to the three most important muscles per pattern. In total, eight muscles were identified: splenius capitis, splenius cervicis, semispinalis capitis, semispinalis cervicis, obliquus capitis inferior, levator scapulae, sternocleidomastoid, and scalenus medius muscles. All muscles were deemed suitable for treatment by BoNT-beginners, and no muscle involved in any of the patterns was excluded.

The CD movement patterns and associated muscles included in Dystonia 3×3 are summarized in Table 1.

### 2.3. Ultrasound Visualization

In the training of BoNT beginners, the Dystonia 3×3 expert consensus strongly recommends ultrasound guidance for all selected muscles, although the superiority of clinical outcomes using ultrasound to guide needle placement versus palpation and anatomical landmarks is still controversial [14,15,16,17,18]. For beginners, however, any visualization of the target muscles offers additional reliability, and today, all training for BoNT in CD treatment includes the use of ultrasound guidance, at least in Germany [19]. Nevertheless, in Dystonia 3×3, the use of ultrasound was only established as mandatory for two of eight muscles: the obliquus capitis inferior and scalenus medius muscles (Figure 2, Figure 3 and Figure 6, respectively). The treatment of these muscles was discussed in earlier publications [20,21]. Some physicians inject the more superficial muscles without technical guidance, still reaching a satisfying clinical outcome. Here, the differentiation of splenius and semispinalis muscles might represent the most important challenge.

After the identification of the target muscles, placement of the ultrasound head for all muscles was determined and is visualized in Figure 1, Figure 2, Figure 3, Figure 4, Figure 5 and Figure 6.

Electromyographic recordings were not included in Dystonia 3×3, as the expert panel considered them unsuitable for beginners. This decision was based, among other considerations, on the lack of standardization of the method. However, experienced users may achieve significant treatment benefits with electromyographic recordings, particularly in patients with CD and tremor or in cases of unclear muscle involvement. Therefore, we will address this method in future publications aimed at experienced users.

### 2.4. Injection Plan

**Figure 7:**
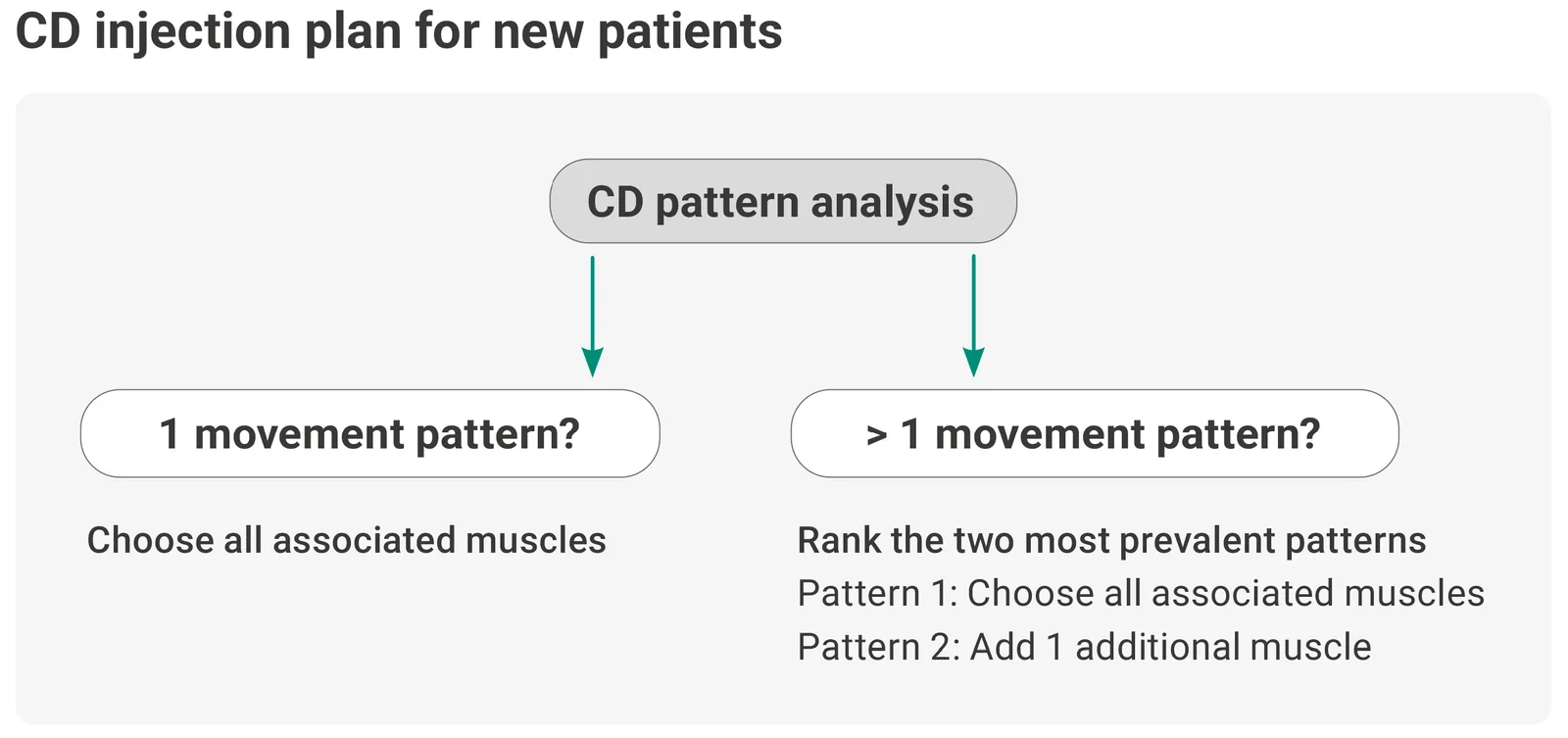
CD injection plan for new patients.

Given the potential clinical complexity of movement patterns in CD, we included a step-by-step approach for the treatment of CD patients naïve to BoNT for beginners. If more than one CD movement is identified in a given patient, the two clinically most prevalent movement patterns are ranked as first and second. Then, all muscles of the first core pattern (two or three) are added to the treatment plan, which is expanded by one additional muscle of the second pattern (see Figure 7). This should be the first muscle on the list that is not already included in the treatment plan. As four of eight muscles in Dystonia 3×3 are associated with more than one movement pattern (splenius capitis, splenius cervicis, levator scapulae, and sternocleidomastoid muscles), a redundancy of muscles is frequent, e.g., causing the combination of torticaput with contralateral laterocaput [10,11]. In this example, the levator scapulae muscle would be added to the treatment plan. Another frequent mixed pattern is torticaput plus retrocaput [11], where the contralateral spinalis capitis muscle, or both semispinalis capitis muscles (bilateral), could be added to the torticaput injection scheme (see Table 1).

For the first treatment of a CD patient naïve to BoNT, Dystonia 3×3 recommends a maximum of three or four muscles for beginners. This results in a maximum of seven individual muscles to be injected in a new CD patient due to the involvement of bilateral muscles in retrocaput, retrocollis, and horizontal tremor (e.g., in horizontal tremor plus collis: splenius capitis (2), sternocleidomastoid (2), obliquus capitis inferior (2), and levator scapulae (1) muscles).

In Dystonia 3×3, the treatment of more complex mixed patterns such as horizontal lateral or sagittal movements (shifts) is not recommended for beginners. In the shifting patterns of CD, identification of the prevalent dystonic element versus compensatory movement is considered unreliable (e.g., laterocollis plus contralateral laterocaput in horizontal shift). Furthermore, in the sagittal variants (anterior and posterior shift), longus capitis and longus colli muscles are probably involved, which are deemed unsuitable for beginners (see above). Also, in some patients, a more detailed differentiation between laterocollis and torticollis is necessary and may lead to the treatment of additional muscles (such as the semispinalis cervicis in torticollis). Future work will address training in BoNT treatment for more complex or mixed CD movement patterns and the use of additional muscles.

### 2.5. BoNT-A Dosing in New Patients

Finally, dose ranges for AboBoNT, OnaBoNT, and IncoBoNT for all muscles were established by the panel (Table 2). Again, dose recommendations were strictly founded both on the beginner in BoNT-A therapy of CD, as well as on a patient naïve to the treatment. For the sternocleidomastoid muscle, a maximum dose in the case of bilateral treatment (in horizontal tremor or retrocaput) was established. This safety measure should avoid adverse events, although there is some uncertainty regarding whether the total dose for the sternocleidomastoid muscle is associated with dysphagia in CD treatment [22]. Dose ranges are required to consider the individual aspects of each patient, foremost the severity of dystonic movements of a given muscle and body weight. Furthermore, we would like to emphasize that dose determination should be based on experience gained through practical teaching courses.

Following an analysis of the patient’s predominant CD movement pattern(s), the injection strategy is adapted according to the complexity of the clinical presentation. If a single movement pattern is identified, all muscles associated with that pattern are selected for injection. If more than one movement pattern is present, the two most prevalent patterns are ranked by clinical relevance. All muscles associated with the primary pattern are selected, while one additional muscle associated with the secondary pattern is included to individualize the injection plan.

In addition, there was strong expert consensus on a low initial dosing in patients naïve to BoNT. Significantly higher BoNT-A doses in long-term treatment (and in more experienced physicians) are optional, as outlined in several treatment guidelines [23,24]. Moreover, a maximum total dose for all selected muscles per patient for the first-ever treatment was recommended: 480 units of AboBoNT and 160 units of OnaBoNT or IncoBoNT, respectively.

## 3. Discussion

Dystonia 3×3 offers a structured and reliable guideline for the beginner on the initial steps of BoNT-A treatment in CD patients. Practitioners can be confident that the muscles selected for the training concept have an impact on the dystonic movement patterns of their patients. All selected muscles were considered suitable for inclusion in the training of new BoNT users. For the obliquus capitis inferior and scalenus medius muscles, ultrasound visualization was considered mandatory because of anatomical complexity.

Particular attention was paid to the graphic illustrations of Dystonia 3×3 to maximize instructiveness and user-friendliness. We are convinced that the extensive discussion among a large group of experts has resulted in a valid and robust guidebook that fills a significant gap in the training of young neurologists.

To provide the beginner with an entry point into the potentially complex treatment of CD, we deliberately simplified several therapeutic variables—the foremost being movement patterns, muscles, and doses. Our work was explicitly based on the Col-Cap-Concept [9,10] without any significant alterations. However, many experts observed that beginners are often intimidated by the larger number of muscles, movement patterns, and their combinations and are hesitant to start therapy. Considering the well-known latency of CD patients until initiation of BoNT toxin treatment [25,26], it is very important to overcome this deficiency.

Several publications have documented that optimizing the BoNT regimen and specifying the injected muscles are essential for improving treatment success and patient satisfaction in CD [27,28]. By restricting the number of muscles per movement pattern, potential overtreatment in CD (as observed by some members of the expert panel) is avoided. As such, the total amount of BoNT might be reduced, and the patients’ feedback on a step-by-step approach will very likely promote the development of an individual injection plan. Finally, the limitation of three muscles in Dystonia 3×3 offers the potential to further delineate the functional anatomy of CD patterns. It might also constitute a valuable data set for future artificial intelligence applications automating CD treatment recommendations [29,30].

The dose range recommendations per muscle in Dystonia 3×3 are lower than real-life experience in most patients, again bearing in mind the beginner in the treatment and a CD patient new to BoNT-A. Significantly higher BoNT-A doses in long-term treatment (and in more experienced physicians) are optional, as outlined in several treatment guidelines [23,24]. Again, it is important to emphasize that dose titration based on the clinical presentation and patient-specific factors remains preferable to using fixed-dose recommendations.

A major limitation of Dystonia 3×3 is the lack of clinical evaluation. Although all recommendations are based on broad expert consensus, we are fully aware that Dystonia 3×3 now requires prospective evaluation, both with regard to its impact on patients’ outcomes and its effectiveness as a training tool. To test the amelioration of CD, a clinical study would have to include a sufficient number of naive patients, as well as evaluations of the motor syndrome and patient-centered outcomes. In addition, the rating of dystonia would have to be independent of the treating physician. Although such a study is in preparation, it should be emphasized again that Dystonia 3×3 was designed exclusively as a starting point for the long-term BoNT treatment of CD patients. A gradual expansion of injection techniques and doses will be covered in subsequent guidelines that will address, among other things, complex and more difficult CD movement patterns, additional muscles, higher doses, and troubleshooting. Therefore, a standalone evaluation of Dystonia 3×3 might fall short of its significance as a training tool.

Assessing the effect of Dystonia 3×3 on the recruitment of new BoNT users, and the quality of training in the therapy of CD, is significantly more challenging. Most importantly, there is no established training concept for BoNT treatment that has been prospectively evaluated and would be a suitable comparison, at least in Germany. Currently, the number of physicians trained using Dystonia 3×3 is monitored, yet any comparison to earlier teaching concepts is vague at best. Our initial feedback to the Dystonia 3×3 training program is very positive, and beginners particularly emphasize the standardization, structuring, and practicality of the guideline.

In addition to the challenges already outlined, we are aware of several limitations of Dystonia 3×3. First of all, our guideline is intended strictly for beginners, which might underwhelm or discourage users who want to tackle more complex and challenging patient cases early on. In this context, the “start low, go slow” principle chosen here could also be criticized, as it may not be adequate for some patients suffering from more severe dystonia. However, we are positive that our upcoming and advanced follow-up guidelines will address this issue. Furthermore, we have to emphasize that beyond BoNT-A, the treatment of CD includes important options regarding motor syndrome (e.g., medication, deep brain stimulation, and physiotherapy), as well as the various non-motor symptoms in CD. These aspects are out of the scope of Dystonia 3×3 and are not addressed here yet are often crucial for our patients.

## 4. Conclusions

The Dystonia 3×3 consensus provides a comprehensive and structured guideline for the BoNT-A treatment of CD, strictly for beginners. It requires prospective evaluation to verify its therapeutic efficacy, to substantiate its simplified anatomical categorization, and to determine how the model should be expanded or adapted for more complex CD presentations.

## 5. Materials and Methods

A panel of German movement disorder specialists was assembled in September 2024, working on the training procedure until December 2025. In total, 17 neurologists (all authors) took part in the study, each holding the certificate “Qualified BoNT Therapy” from the BoNT Working Group of the German Society of Neurology. The panel has extensive experience in BoNT-A therapy in CD, with a total of more than 220,000 individual treatments.

Panel discussions on all treatment steps were free of preset limitations. Beforehand, however, a framework of results was defined as *eligible for beginners in BoNT-A treatment* of CD, considering the target audience of the guideline. Furthermore, the selection of muscles was restricted to the *minimum required to successfully treat CD* for each movement pattern selected. These prerequisites of the guideline were presented by SP and DW in the first group discussion.

Panel decisions were established over 5 review phases. Expert consensus was defined as 16/17 (94%) or 17/17 (100%) agreement, respectively. In the first phase, key elements of the guideline were defined through group discussion, including the target audience, measures to simplify training, components of BoNT treatment to be included, movement patterns and associated muscles, and the use of ultrasound imaging. The discussion was based on material prepared by SP and DW, as well as specifications of the Col-Cap pattern [9] and subsequent elaborations and guidelines [1,8,14,31,32]. In the second phase, results of the initial discussion were documented in writing and visual form and presented to the co-authors online for discussion and revision. Once the target muscles had been finalized, another online round was conducted to determine dose recommendations. In the fourth phase, remaining open questions were addressed in a face-to-face discussion, again facilitated by SP and DW. This was followed by the finalization of the training concept in the form of a summary text. In the final two review phases, the summary text underwent online review and revision, ultimately resulting in the present manuscript.

All patients (E.W., M.G., M.G., R.L.) and authors (F.M., S.P.) gave consent for publication of their images in the manuscript.

## Figures and Tables

**Table 1:** Dystonia 3×3—CD movement patterns and associated muscles.

CD Pattern	Associated Muscles	Location	Figure
Laterocaput	Splenius capitis	Ipsilateral	Figure 1
	Levator scapulae	Ipsilateral	
	Sternocleidomastoid	Ipsilateral	
Torticaput	Splenius capitis	Ipsilateral	Figure 2
	Sternocleidomastoid	Contralateral	
	Obliquus capitis inferior ^2^	Ipsilateral	
Collis ^1^	Levator scapulae	Ipsilateral	Figure 3
	Splenius cervicis	Ipsilateral	
	For laterocollis, add scalenus medius ^2^	Ipsilateral	
Retrocaput	Semispinalis capitis	Bilateral	Figure 4
	Splenius capitis	Bilateral	
	Sternocleidomastoid	Bilateral	
Retrocollis	Semispinalis cervicis	Bilateral	Figure 5
	Splenius cervicis	Bilateral	
Horizontal tremor	Splenius capitis	Bilateral	Figure 6
	Sternocleidomastoid	Bilateral	
	Obliquus capitis inferior ^2^	Bilateral	

^1^ Subsumes torticollis and laterocollis. ^2^ Ultrasound visualization is mandatory for muscle identification and injection plan for new CD patients.

**Table 2:** Dystonia 3×3—dose ranges for naïve CD patients ^1^.

Muscle	Dose Ranges (Units)	
	AboBoNT	OnaBoNT IncoBoNT
Splenius capitis	40–120	15–40
Splenius cervicis	40–120	15–40
Semispinalis capitis	40–120	15–40
Semispinalis cervicis	40–100	15–30
Obliquus capitis inferior	40–120	15–40
Levator scapulae	40–120	15–40
Sternocleidomastoid ^2^	40–100	15–30
Scalenus medius	20–100	10–30
First treatment maximum	480	160

^1^ When choosing the dose in the range, consider the patient’s characteristics such as severity of dystonia and body weight. ^2^ For injection of both sternocleidomastoid muscles (bilateral), a combined dose not higher than 180 units AboBoNT, or 60 units OnaBoNT or IncoBoNT, is recommended.

## Data Availability

The original contributions presented in this study are included in the article. Further inquiries can be directed to the corresponding author(s).

## Abbreviations

The following abbreviations are used in this manuscript:

CD	Cervical dystonia
BoNT-A	Botulinum neurotoxin type A
AboBoNT-A	AbobotulinumtoxinA
OnaBoNT-A	OnaBotulinumtoxinA
IncoBoNT-A	IncobotulinumtoxinA
